# Sleep Architecture and Microstructure in Childhood Absence Epilepsy: Clinical and Neurophysiological Perspectives

**DOI:** 10.3390/jcm15093454

**Published:** 2026-05-01

**Authors:** Małgorzata Jączak-Goździak, Marcin Żarowski

**Affiliations:** Department of Developmental Neurology, Poznan University of Medical Sciences, 60-355 Poznan, Poland

**Keywords:** childhood absence epilepsy, sleep architecture, sleep microstructure, sleep spindles, thalamocortical networks, cognitive impairment

## Abstract

Childhood absence epilepsy (CAE) is one of the most common epilepsy syndromes in childhood and has traditionally been regarded as a condition with a favorable neurological prognosis. However, increasing evidence suggests that CAE is associated with functional disturbances in neuronal networks that extend beyond seizure generation and may involve sleep and wakefulness regulation. Methods: This narrative mini-review summarizes and critically discusses current clinical and neurophysiological evidence regarding alterations in sleep architecture and sleep electroencephalographic (EEG) microstructures in children with CAE, based on a focused analysis of selected clinical and observational studies. Results: The available data suggest that children with CAE, particularly before treatment initiation, may exhibit sleep macrostructure abnormalities, including reduced total sleep time, prolonged rapid eye movement sleep latency, increased arousal frequency, and decreased sleep efficiency. In addition, changes in sleep microstructure have been reported, most notably reduced sleep spindle density during stage-N2 sleep, especially in patients with concomitant cognitive impairment. These findings may reflect alterations in thalamocortical network function, although current evidence remains limited and heterogeneous. Conclusions: Sleep disturbances appear to represent an important component of the clinical phenotype of childhood absence epilepsy. Assessing the sleep architecture and sleep EEG microstructure, particularly sleep spindles, may provide insights into network dysfunction and cognitive vulnerability; however, further studies are needed to clarify their clinical utility.

## 1. Introduction

Childhood absence epilepsy (CAE) is one of the most common epilepsy syndromes in childhood and belongs to the group of idiopathic generalized epilepsies. It is characterized by frequent, brief episodes of impaired awareness associated with generalized, bilateral, synchronous spike–wave discharges on electroencephalography (EEG), typically at approximately 3 Hz. According to the current International League Against Epilepsy (ILAE) classification, CAE is an age-dependent epilepsy syndrome with onset usually between 4 and 10 years of age, a normal neurological examination at diagnosis, and an absence of structural brain abnormalities [1,2,3].

For decades, CAE has been considered a relatively benign epilepsy syndrome, largely due to its high rate of seizure remission during adolescence and favorable long-term seizure outcome in the majority of patients [4,5,6,7]. This perception has shaped clinical practice, leading to a primary focus on seizure control and pharmacological management. However, increasing evidence suggests that this concept may be incomplete, as CAE can be associated with persistent neuropsychological and functional consequences.

Several studies have demonstrated that children with CAE frequently exhibit deficits in attention, executive functions, processing speed, and academic performance, even at disease onset and before treatment initiation [8,9]. Importantly, these cognitive difficulties may persist despite adequate seizure control and normalization of routine awake EEG recordings, suggesting that seizure suppression alone may not fully capture disease burden. Such findings suggest that CAE may involve broader disturbances of brain network function rather than being limited to transient epileptic events.

From a pathophysiological perspective, CAE has been proposed as “system epilepsy”, involving abnormal interactions within thalamocortical circuits and extended brain networks [4,10]. These networks are thought to play a central role not only in generating generalized spike–wave discharges but also in regulating vigilance states, sleep architecture, and physiological oscillations relevant for cognitive development. Consequently, sleep may represent a valuable window into the functional integrity of these networks.

A complex, bidirectional relationship links sleep and epilepsy. Sleep modulates epileptic activity, while epileptic discharges and seizures may disrupt sleep continuity and organization [11,12,13]. In idiopathic generalized epilepsies, including CAE, epileptic activity shows a strong dependence on vigilance states, with increased occurrence during non-rapid eye movement (NREM) sleep and during transitions between sleep and wakefulness [12]. These observations suggest that sleep-related network dynamics may play an important role in the pathophysiology of CAE.

Sleep disturbances in CAE have historically received less attention than seizure semiology or awake EEG findings, despite the close relationship between sleep and epileptic activity. Routine clinical evaluation often relies on short awake EEG recordings, while sleep EEG or polysomnography is typically reserved for selected cases. As a result, subtle abnormalities in sleep architecture and microstructure may remain undetected in many patients.

Recent investigations have begun to address this gap by examining sleep in children with CAE. Available studies suggest that both sleep macroarchitecture and sleep microstructure may be altered, even in drug-naïve patients, and may be associated with cognitive impairment and network dysfunction [8,14]. Of particular interest are sleep spindles—transient oscillatory events generated within thalamocortical circuits during stage-N2 sleep—which play a key role in synaptic plasticity, memory consolidation, and neurodevelopment [15]. Disrupting these physiological oscillations may therefore have implications for cognitive outcomes in CAE.

Although interest in this area has increased in recent years, the role of sleep architecture and sleep EEG microstructure in childhood absence epilepsy remains incompletely understood, and available findings are heterogeneous and not systematically integrated.

Therefore, this mini-review aims to provide a structured and critical overview of current clinical and neurophysiological evidence regarding alterations in sleep architecture and sleep EEG microstructure in childhood absence epilepsy, with particular emphasis on their potential clinical relevance and underlying network mechanisms.

## 2. Methods

This narrative mini-review was based on a focused literature search conducted using the PubMed database from 2000 to January 2026. The search employed combinations of the following keywords: “childhood absence epilepsy”, “sleep”, “sleep architecture”, “sleep spindles”, and “EEG”.

Only articles published in English were considered. We included original clinical studies, observational studies, and relevant review articles addressing sleep architecture or sleep EEG microstructure in childhood absence epilepsy.

Study selection was based on their relevance to the topic and their contribution to understanding thalamocortical mechanisms and sleep-related network dysfunction in childhood absence epilepsy. Given the narrative nature of the review, no formal systematic selection protocol was applied.

## 3. Sleep Architecture in Childhood Absence Epilepsy

Sleep architecture refers to the global organization of sleep, encompassing both its quantitative and temporal structure. It includes parameters such as total sleep time, sleep efficiency, sleep latency, and the distribution and cyclic organization of the rapid eye movement (REM) and non-rapid eye movement (NREM) sleep stages (N1, N2, and N3) throughout the sleep period. These stages recur in a characteristic cyclic pattern across the night, reflecting the overall structure and continuity of sleep [16].

In children with CAE, sleep macroarchitecture has been less extensively studied than seizure semiology or awake EEG features; however, the available evidence suggests that abnormalities may be present in a subset of patients.

Prospective polysomnographic studies in relatively small cohorts of drug-naïve children with CAE have demonstrated alterations in global sleep organization, including reduced total sleep time, decreased sleep efficiency, increased frequency of nocturnal awakenings, and prolonged REM sleep latency [14]. Importantly, these abnormalities were observed before the initiation of antiepileptic treatment, suggesting that disturbed sleep architecture may represent an intrinsic feature of the disorder rather than a secondary effect of medication or chronic seizure burden.

Longitudinal observations provide further insight into the relationship between seizure control and sleep architecture. An improvement in sleep continuity and efficiency has been reported following effective treatment, as well as a reduction in epileptic activity during both wakefulness and sleep [17]. Nevertheless, normalizing macrostructural sleep parameters does not necessarily imply complete restoration of physiological sleep-related network function, particularly at the microstructural level.

Not all studies have identified pronounced abnormalities of sleep macroarchitecture in CAE. In some cohorts, conventional sleep stage distribution and total sleep time were largely preserved despite the presence of epileptic discharges during sleep [8]. This apparent discrepancy highlights the heterogeneity of CAE. It may reflect differences in disease duration, cognitive profile, treatment status, and methodological approaches to sleep assessment, as well as the limited sensitivity of macrostructural measures [8,14]. These inconsistencies suggest that macrostructural sleep alterations in CAE are not uniform across studies and should be interpreted with caution.

Importantly, sleep recordings may reveal greater electroencephalographic heterogeneity in CAE than wake recordings alone. Pretreatment sleep EEG studies in pediatric patients have demonstrated focal or asymmetric interictal discharges and polyspike activity in a subset of patients who otherwise meet diagnostic criteria for generalized epilepsy [18]. These findings may suggest that sleep unmasks network-level instabilities that remain undetected during routine awake EEG.

The occurrence of absence seizures during sleep, although relatively uncommon, further underscores the relevance of sleep architecture in CAE. Such seizures tend to occur during light NREM sleep and periods of sleep instability and have been associated with poorer seizure control in some patients [19].

Collectively, these observations suggest that sleep macroarchitecture provides important contextual information for understanding epileptic activity in CAE but may lack sensitivity for detecting subtle network dysfunction (Table 1).

## 4. Sleep Microstructure and Thalamocortical Mechanisms in Childhood Absence Epilepsy

Sleep microstructure refers to transient electroencephalographic (EEG) phenomena and fine-grained oscillatory patterns occurring within sleep stages, including sleep spindles, slow oscillations, K-complexes, and arousal-related events. These features reflect dynamic interactions within thalamocortical networks and may provide a sensitive marker of functional network integrity.

Early electroencephalographic studies demonstrated a close temporal relationship between spike–wave discharges (SWDs) and transitions across vigilance states, supporting the concept of a sleep–wake continuum rather than discrete pathological events [12]. In childhood absence epilepsy (CAE), epileptic discharges preferentially emerge during light non-rapid eye movement (NREM) sleep and at transitions between sleep stages, suggesting a role of sleep instability in seizure generation [12,20,21].

A growing body of evidence suggests that SWDs may be linked to the microstructure of sleep, particularly to transient fluctuations in arousal level. They have been reported to occur more frequently during unstable brain states, such as micro-arousals and cyclic alternating pattern (CAP) phase A, especially subtype A1, which is characterized by synchronized slow-wave activity. However, evidence on childhood absence epilepsy remains limited [12,21]. These observations suggest that absence seizures may be facilitated by dynamic fluctuations in cortical excitability rather than by stable sleep stages alone.

Sleep spindles constitute a core element of sleep microstructure and are generated through coordinated interactions between thalamocortical relay neurons and inhibitory neurons of the thalamic reticular nucleus. Historically, SWDs were hypothesized to represent a pathological transformation of sleep spindles. However, subsequent experimental and clinical studies indicate that although both phenomena arise within overlapping thalamocortical networks, they differ in their sites of initiation, regulatory mechanisms, and functional roles [19]. In particular, sleep spindles are primarily generated within thalamic circuits, whereas SWDs are thought to be initiated in cortical regions and subsequently engage thalamocortical loops [8,18,21,22].

Recent clinical studies have further refined this framework by demonstrating reduced sleep spindle density and duration in children with CAE, particularly in those with coexisting cognitive impairment [8]. These findings suggest that alterations in sleep microstructure may be associated with cognitive dysfunction and reflect underlying thalamocortical network dysfunction. However, these observations should be interpreted as associations rather than direct evidence of causal mechanisms linking sleep spindle abnormalities with cognitive impairment.

Contemporary models of absence epilepsy emphasize dynamic and spatially structured thalamocortical interactions rather than uniform global hypersynchrony [23]. Neurophysiological studies indicate that generalized ictal discharges exhibit non-uniform spatiotemporal propagation patterns, suggesting organized network dynamics rather than diffuse activation [22]. Furthermore, detailed sleep EEG analyses indicate that both focal and generalized SWDs are topographically structured and may be associated with specific phases of NREM sleep microstructure, particularly during the early sleep cycles [21].

While the involvement of thalamocortical circuits in absence epilepsy is well established, the relationship between sleep microstructure alterations and cognitive dysfunction remains largely conceptual and requires further investigation.

## 5. Clinical Implications

Sleep disturbances in childhood absence epilepsy may represent an important component of the disorder and reflect underlying network dysfunction. Routine clinical evaluation may benefit from greater attention to sleep-related symptoms and, in selected cases, from the use of sleep EEG or prolonged video EEG monitoring.

In clinical practice, sleep EEG may be particularly useful in patients with atypical clinical features, cognitive difficulties, or suboptimal treatment response, where additional information beyond standard awake EEG recordings may be required. Importantly, sleep EEG is often more feasible and technically easier to perform in children than full polysomnography, which may further support its broader clinical applicability.

Most EEG predictors of treatment response and long-term outcomes are derived predominantly from awake recordings. In contrast, sleep EEG remains relatively underutilized, despite emerging evidence of its potential prognostic value in idiopathic generalized epilepsies [24,25].

An assessment of sleep microstructure, including sleep spindle characteristics, may provide complementary information beyond standard EEG measures. However, its role in routine clinical practice remains to be established, and further prospective studies are needed to determine its value in identifying patients at risk for persistent neuropsychological difficulties.

## 6. Future Perspectives

Future studies may benefit from the systematic integration of standardized sleep assessments into the clinical evaluation of childhood absence epilepsy, particularly in newly diagnosed and drug-naïve patients. Advances in quantitative EEG analysis and automated spindle detection offer promising tools to objectively assess sleep microstructure and thalamocortical network function.

From a broader conceptual perspective, CAE has been proposed to represent a disorder of the sleep–wake system rather than a purely seizure-based condition, aligning with the concept of “system epilepsies” [26]. However, this framework remains largely theoretical and requires further validation in clinical studies.

A better understanding of sleep-related network alterations may help develop more individualized management strategies. These may include approaches targeting sleep quality and circadian regulation; however, their clinical applicability remains to be established.

## 7. Conclusions

Childhood absence epilepsy is increasingly recognized as a condition involving distributed thalamocortical networks rather than being defined solely by transient absence seizures. Accumulating clinical and neurophysiological evidence suggests that alterations in sleep architecture, particularly sleep microstructure, may be important components of the clinical phenotype of CAE.

While sleep macroarchitecture abnormalities appear to be variable and often modest, changes in sleep microstructure—particularly reduced sleep spindle density—may more sensitively reflect underlying network dysfunction and cognitive vulnerability. However, current evidence remains limited and heterogeneous, and further studies are needed to clarify these relationships.

Incorporating sleep-focused evaluations into clinical practice may improve disease characterization and risk stratification. Nevertheless, the clinical utility of such approaches, including microstructures, requires further validation in prospective studies.

## Figures and Tables

**Table 1:** Alterations of sleep macroarchitecture in childhood absence epilepsy.

Study	Study Design	Patient Group	Method	Main Findings
Dinopoulos et al., 2018 [14]	Prospective case–control study	28 drug-naïve children (21 girls), mean age 90.1 months, diagnosed with CAE	Routine EEG and overnight polysomnography at diagnosis and 12 months post-treatment	Absolute control of absences improved total sleep time and REM sleep and reduced arousal. Persistent epileptic activity was associated with sleep instability. No correlation found between initial high epileptic load and drug resistance.
Zhang et al., 2023 [8]	Comparative study	29 drug-naïve CAE patients (mean age 8 years) vs. 30 healthy controls	Overnight video EEG and Wechsler Intelligence Scale for Children-Fourth Edition (WISC-IV)	Preserved macrostructure. Microstructural abnormalities: CAE patients showed significantly lower sleep spindle (SS) density and duration in N2 sleep. Deficits in SS density correlated with impaired cognitive function (FSIQ < 70), suggesting SSs as a potential biomarker for cognitive impairment.
Edizer et al., 2023 [17]	Retrospective study	62 drug-naïve patients (32 CAE, 30 JAE)	Awake and sleep EEG recordings at baseline and 12-month follow-up	JAE patients exhibited more disorganized discharges and polyspikes in sleep than CAE patients. Focal-onset generalized spike–wave discharges and high discharge density in baseline sleep EEGs predicted poor medication response in JAE. Improvement in sleep EEG after treatment.
Chen et al., 2022 [19]	Case report	1 patient (female, 7–8 years old), drug-resistant CAE	Overnight video EEG recording (10 h sleep) and C-WISC	First report of absence seizures occurring during non-REM stage 2 sleep in refractory CAE. Findings suggest sleep-based absence seizures may indicate drug resistance and poor prognosis; cognitive decline was noted (FSIQ dropped from 100 to 81).

Abbreviations: CAE—childhood absence epilepsy; JAE—juvenile absence epilepsy; EEG—electroencephalography; REM—rapid eye movement sleep.

## Data Availability

No new data were created or analyzed in this study.

## Abbreviations

The following abbreviations are used in this manuscript:

CAE	Childhood absence epilepsy
JAE	Juvenile absence epilepsy
EEG	Electroencephalography
PSG	Polysomnography
REM	Rapid eye movement sleep
NREM	Non-rapid eye movement sleep
SWD	Spike–wave discharge
